# Effects of Gestational Diabetes Mellitus and Selenium Deficiency on the Offspring Growth and Blood Glucose Mechanisms of C57BL/6J Mice

**DOI:** 10.3390/nu15214519

**Published:** 2023-10-25

**Authors:** Wenhui Xu, Jiayu Gong, Yifei Chen, Yiru Chen, Shutong Chen, Yanyan Wu, Yuan He, Chenxu Li, Haitao Yu, Lin Xie

**Affiliations:** 1 School of Public Health, Jilin University, Changchun 130012, China; xuwh20@mails.jlu.edu.cn (W.X.); gongjy21@mails.jlu.edu.cn (J.G.); cyf21@mails.jlu.edu.cn (Y.C.); chenst22@mails.jlu.edu.cn (S.C.); wuyy22@mails.jlu.edu.cn (Y.W.); heyuan22@mails.jlu.edu.cn (Y.H.); chenxu@jlu.edu.cn (C.L.); 2 Clinical Nutrition Department, Third Hospital of Jilin University, Changchun 130032, China; 18843113308@163.com

**Keywords:** gestational diabetes mellitus, offspring, glucose, growth and development

## Abstract

This study aimed to explore the effects and mechanisms of maternal gestational diabetes mellitus (GDM) and selenium (Se) deficiency on the growth and glucose metabolism of offspring. Female C57BL/6J mice were divided into four groups as follows: a control group, a GDM group, a Se deficiency group, and a GDM with Se deficiency group. GDM animal models were established via S961. Pregnant mice fed their offspring until weaning. Then, offspring continued to be fed with a basic diet until adulthood. Body weight and fasting blood glucose were measured weekly. Se content, oxidative stress indicators, and the protein expression of the PI3K/Akt signaling pathway were detected. GDM increased susceptibility to obesity in lactating offspring, with gender differences observed in adult offspring. The effect of Se deficiency on SOD activity only appeared in female offspring during adulthood but was shown in male offspring during weaning though it disappeared during adulthood. GDM and Se deficiency increased the risk of abnormal glucose metabolism in female offspring from weaning to adulthood but gradually decreased in male offspring. The influence on the expression of PI3K/Akt signaling pathway-related proteins showed the same trend. GDM and Se deficiency affected the growth and glucose metabolism of offspring through oxidative stress and PI3K/Akt signaling pathway-related proteins, and gender differences existed.

## 1. Introduction

Gestational diabetes mellitus (GDM) refers to pregnant women who have normal glucose metabolism or potentially impaired glucose tolerance before pregnancy but have symptoms or are diagnosed during pregnancy [1,2]. The prevalence rate of GDM is high in Asian countries and regions [3,4,5]. This disease not only impacts the health of pregnant women but also adversely affects them. GDM is a risk factor for poor offspring development and the occurrence of metabolic-related diseases from birth to adulthood.

Selenium (Se) is an essential trace element in the human body. According to the 2016 Chinese Dietary Guidelines for Residents, the recommended dietary intake for Se is 65 µg/d for pregnant women and 78 µg/d for lactating mothers [6]. Within the global territory, China is a region with low Se levels and extremely low Se levels from northeast to southwest. The main function of Se is selenoprotein in the form of an enzyme. With further research on the relationship between Se and diseases, some scholars have found that Se deficiency is associated with an increased risk of GDM [7,8,9,10,11,12,13,14]. GDM may be a disease due to multiple factors, but its exact pathogenesis is still unclear [15]. Glutathione peroxidase (GSH-Px) and other selenoproteins related to oxidative stress are essential for GDM [16].

The PI3K/Akt signaling pathway is associated with Se deficiency and oxidative stress [17,18]. PIP5K1A is a kind of phosphatidylinositol phosphokinase and a major regulator of many biological processes, such as cell differentiation and migration. It acts upstream of the PI3K/Akt signaling pathway [19]. NOX is the main enzyme produced by non-mitochondrial excess reactive oxygen species (ROS) in cells. The downstream signal of NOX1 is Akt, which participates in tissue oxidative stress by inhibiting the activation of Akt phosphorylation [20,21]. GDM is associated with Se deficiency, insulin resistance, and oxidative stress and could be related to the expression of PI3K/Akt signaling pathway-related proteins.

The current study aims to explore the relationship between GDM and Se deficiency and its possible mechanism, provide a basis for reducing the incidence rate of GDM in the future, and propose new ideas for treating and preventing GDM and the healthy growth of offspring.

## 2. Materials and Methods

Eighty female and forty male C57BL/6J mice (Skbex Biotechnology, Henan, China) were maintained in standard housing conditions (12 h light/dark cycle and 22 ± 2 °C) at the School of Public Health, Jilin University. Mice had ad libitum access to tap water and a basic diet (H10010, Huafukang Bioscience Co., Ltd., Beijing, China, Se content was 0.2 mg/kg, and the composition of the basic diet is shown in Supplementary Table S1) or a low-Se diet (Se content was 0.02 mg/kg, other components were the same as H10010). Groups were age-matched (6–8 weeks). They were randomly divided into four groups (n = 20/group). Two groups were given the basic diet, and the other two were given a low-Se diet. After 4 weeks, fasting blood glucose (FBG) was measured as the baseline blood glucose, and then females and males were mated at a 2:1 rate. The day of vaginal plug observation was recorded as Gestation Day 1 (GD 1), and pregnant mice were included. After pregnancy, FBG was measured on GD 7 (before treatment). FBG in the tail vein of pregnant mice was measured using a blood glucose meter (Abbott, Chicago, IL, USA). A hypodermic injection of PBS or S961 (Jiepeptide Biotechnology Co., Ltd., Nanjing, China) began on GD 7; two groups were injected with PBS, and the other two groups were injected with S961. The four groups were as follows: the control group (CON): basic diet + PBS; GDM group (GDM): basic diet + S961; the Se deficiency group (LSe): low-Se diet + PBS; GDM with Se deficiency group (LSe-GDM): low-Se diet + S961. The S961 peptide was dissolved in PBS. S961 was injected once a day at 18:00 in the GDM and LSe-GDM groups with a 20 nmol/kg·bw dose. The same dose of PBS was injected subcutaneously in CON and LSe groups until GD 17 or delivery. All animal procedures were approved by the Ethics Committee of Jilin University School of Public Health (license 2021-04-06).

The oral glucose tolerance test (OGTT) was performed on GD 14, where the FBG of pregnant mice was measured after 5 h of fasting, which was recorded as 0 min FBG. Glucose of 1.2 g/kg·bw was given using a gavage. Blood glucose levels were measured at 30, 60, 120, and 180 min after gavage. Based on the diagnostic criteria of human GDM, FBG, the 1 h blood glucose and 2 h blood glucose thresholds were 5.1 mmol/L, 10.0 mmol/L, and 8.5 mmol/L, respectively. Due to the influence of animal experimental blood glucose values on glucose concentration, detection methods, and procedures, this study combined the blood glucose levels at various time points of OGTT with the existence of statistical differences in the area under the blood glucose curve between the GDM group and non-GDM group to determine whether the experimental animals developed insulin resistance. According to previous studies, a dietary Se content < 0.05 mg/kg is defined as Se deficiency [22]. If Se levels or GSH-Px activities in the two Se deficiency groups are significantly lower than those in the two basic diet groups, it can be demonstrated that the Se deficiency model is successfully established.

On GD 18, some pregnant mice were weighed and euthanized; blood was drawn from the heart, and their serum, liver, kidney, pancreas, and other tissues were collected. The placenta and fetal mice tissues from the fetal sac were separated; the weight of each tissue was measured and recorded, and the organ coefficient was calculated (organ coefficient = organ weight/body weight × 100%). These tissues were stored at −80 °C. Others delivered and fed their offspring until weaning (4 weeks old). Some of the weaning offspring were euthanized, and others were given a basic diet until adulthood (8 weeks old). The weaning and adult offspring were subjected to the same treatment.

The serum insulin levels of pregnant mice on GD 18 were detected using an Enzyme-linked Immunosorbent Adsorption Test kit (Youxuanshengwu, Shanghai, China). The Se level in the kidney was detected using atomic fluorescence spectroscopy (AFS). According to Supplementary Table S2, the solution was pipetted into a 10 mL volumetric flask that was mixed well; the fluorescence intensity was measured on the machine to draw the Se standard curve. The sample pre-treatment in this experiment was wet digestion and the Se content of the sample was calculated using the formula: X = (C − C_0_) × V/(m × 1000), where X represents the Se content of the sample (mg/kg); C represents the concentration of the sample digestive solution (ng/mL); C_0_ represents the concentration of blank digestion solution in the sample (ng/mL); m represents the weight of the sample (g); and V represents the volume of the sample digestion solution (mL). The GSH-Px, superoxide dismutase (SOD), and malonaldehyde (MDA) test kits (Nanjing Jiancheng Bioengineering Institute, Nanjing, China) were used to measure oxidative stress indicators in liver tissue. The weaning and adult offspring were subjected to the same treatment.

Protein samples were extracted from the liver or placenta homogenate in a 1% protease inhibitor (PMSF, Beyotime Biotechnology, Shanghai, China). The protein concentration was quantified using the BCA assay system (Beyotime Biotechnology, Shanghai, China). Proteins were analyzed by 8% or 12% FuturePAGE^TM^ and transferred to Polyvinylidene Fluoride (PVDF) membranes using the wet transfer unit. The PVDF was immersed in a blocking buffer (TBST, 0.1% Tween-20) with 5% non-fat milk for 2 h and then incubated with primary antibody overnight at 4 °C. After washing with a blocking buffer; the blots were incubated with an appropriate HRP-conjugated secondary antibody at room temperature for 45 min and washed again using a blocking buffer. Immunoreactive proteins were detected using enhanced chemiluminescence substrate reactions. PI3K, Akt, NOX1, and PIP5K1A antibodies were purchased from Proteintech Group. Supplementary Table S3 shows the specific antibody dilution.

BONC DSS Statistics 25.0 software was used for statistical analysis. Normal data were represented by the Mean ± SD, and abnormal data were expressed by the median (P_25_, P_75_). Two-way ANOVA was performed for interaction analysis. One-way ANOVA was conducted to compare normal distribution data; the abnormal distribution data were compared using the Kruskal–Wallis test. *p* < 0.05 indicated statistically significant differences. Image J 1.53a was used to analyze Western blot data, and Graphpad Prism 5.0 software was used to draw a statistical graph.

## 3. Results

### 3.1. Effects of GDM and Se Deficiency on the Growth and Development of Offspring

**Figure 1:**
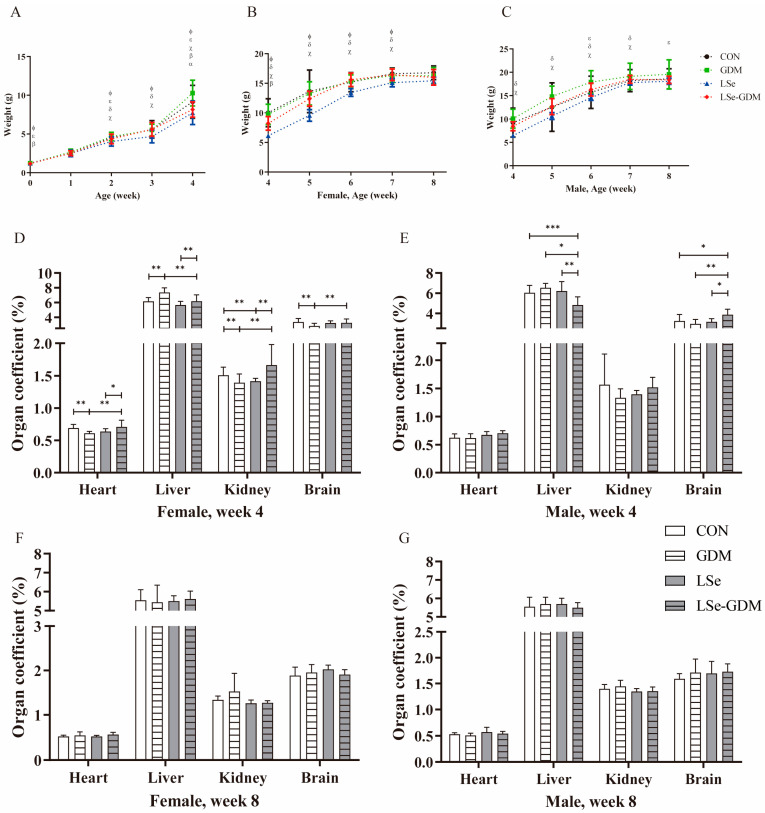
The effects of GDM and Se deficiency on the growth and development of offspring. (**A**) The body weight of offspring from birth to week 4; (**B**) The body weight of female offspring from weeks 4 to 8; (**C**) The body weight of male offspring from weeks 4 to 8; ^α, β, χ, δ, ε, Φ^ indicate *p* < 0.05, ^α^ CON vs. GDM, ^β^ CON vs. LSe-GDM, ^χ^ GDM vs. LSe, ^δ^ LSe vs. LSe-GDM, ^ε^ GDM vs. LSe-GDM, ^Φ^ CON vs. LSe; CON, basic diet + PBS injection; GDM, basic diet + S961 injection; LSe, low-Se diet + PBS injection; LSe-GDM, low-Se diet + S961 injection. (**D**) The organ coefficient of female offspring in the 4th week; (**E**) The organ coefficient of male offspring in the 4th week; (**F**) The organ coefficient of female offspring in the 8th week; (**G**) The organ coefficient of male offspring in the 8th week. * indicate *p* < 0.05, ** indicate *p* < 0.01, *** indicate *p* < 0.001.

The establishment of GDM and Se-deficient animal models is shown in Supplementary Figures S1 and S2. The weight changes in the offspring from birth to weaning are shown in Figure 1A. The birth weights in the CON group were significantly higher than those in the LSe and LSe-GDM groups, and the birth weights in the GDM group were significantly higher than those in the LSe-GDM group. No differences were observed in weight during the first week among the four groups. The 2-week-old body weights in the GDM, LSe-GDM, and CON groups were significantly higher than those in the LSe group, while the GDM group had a significantly higher body weight than the LSe-GDM group. Body weights in the third week in the GDM, LSe-GDM, and CON groups were significantly higher than those in the LSe group. In the fourth week, the body weight in the GDM group was significantly higher than that in the CON group, while the body weights in the GDM and CON groups were significantly higher than those in the LSe-GDM group; the body weights in the GDM and CON groups were significantly higher than those in the LSe group.

The offspring were weaned and fed a basic diet as they grew. The effects of GDM and a low-Se diet on the weight of female mice decreased (Figure 1B). From weeks 4 to 7, body weight in the CON, GDM, and LSe-GDM groups was significantly higher than that in the LSe group; in the 4th week, body weight in the CON group was significantly higher than that in the LSe-GDM group. Weight gain tended to stabilize in adulthood in both the basic feed and Se deficiency groups, and no difference was observed in weight among the four groups on the 8th week.

Male offspring body weight (Figure 1C) in the GDM and LSe-GDM groups was significantly higher than that in the LSe group from weeks 4 to 7, while body weight in the GDM group was significantly higher than in the LSe-GDM group at weeks 6 and 8. After weaning, GDM increased male offspring weight.

Female and male offspring showed statistically significant differences in organ coefficients during the weaning period. There were statistically significant differences in the heart (*p* = 0.004), liver (*p* < 0.001), kidney (*p* < 0.001), and brain coefficients (*p* = 0.033) among the four groups of female offspring (Figure 1D). Meanwhile, there were statistically significant differences in the liver (*p* = 0.001) and brain coefficients (*p* = 0.035) among the four groups of male offspring (Figure 1E). There were also no differences in these four organ coefficients among the groups in adult female and male offspring (Figure 1F,G).

### 3.2. Effect of GDM and Se Deficiency on Blood Glucose in Offspring

The FBG levels in female and male offspring were measured at weaning. The FBG levels in the GDM and LSe-GDM groups were significantly higher than those in the CON and LSe groups in female offspring (Table 1). The FBG levels in the LSe group were significantly higher than those in the CON group during the seventh week (*p* = 0.002), while FBG levels in the GDM group were significantly higher than those in the CON group in the eighth week (*p* = 0.007).

FBG levels in the CON (*p* = 0.003) and GDM (*p* = 0.001) groups were significantly higher than those of the LSe group for male offspring (Table 1). The effects of maternal GDM and Se deficiency on male offspring were reduced after weaning.

Factorial analysis revealed that GDM induced an increase in FBG levels during the fourth (*p* < 0.001) and eighth week (*p* = 0.021) but had no effect on FBG levels from weeks 5 to 7 for female offspring. Maternal Se deficiency increased FBG levels at week 7 (*p* = 0.023) but had no effect on FBG levels at weeks 4, 5, 6, and 8. The interaction between GDM and Se deficiency had an effect on FBG at weeks 5 (*p* = 0.033) and 7 (*p* = 0.019) but had no effect on FBG during the 4th, 6th, and 8th weeks. Se deficiency in male offspring resulted in a decrease in FBG on the 4th week (*p* = 0.001) but had no effect on FBG from weeks 5 to 8.

### 3.3. Effects of GDM and Se Deficiency on Oxidative Stress in Offspring

**Figure 2:**
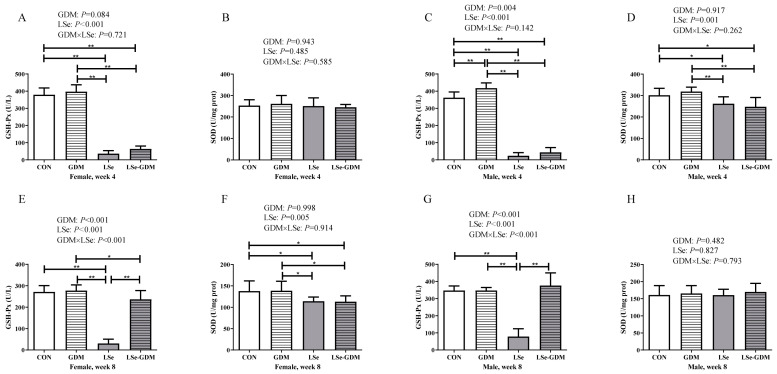
The effects of GDM and Se deficiency on oxidative stress in offspring. (**A**) The liver GSH-Px of female offspring during the 4th week; (**B**) The liver SOD of female offspring during the 4th week; (**C**) The liver GSH-Px of male offspring during the 4th week; (**D**) The liver SOD of male offspring during the 4th week; (**E**) The liver GSH-Px of female offspring during the 8th week; (**F**) The liver SOD of female offspring during the 8th week; (**G**) The liver GSH-Px of male offspring during the 8th week; (**H**) The liver SOD of male offspring during the 8th week; * indicate *p* < 0.05, ** indicate *p* < 0.01.

Liver GSH-Px activity in the LSe and LSe-GDM groups was significantly lower than that in the CON and GDM groups for female offspring during weaning (Figure 2A). Liver GSH-Px activity in the LSe and LSe-GDM groups was significantly lower than that in the CON and GDM groups, and GSH-Px activity in the CON group was significantly lower than that in the GDM group for male offspring during weaning; liver SOD activity in the LSe and LSe-GDM groups was significantly lower than that in the CON and GDM groups (Figure 2C,D).

A decrease in GSH-Px activity owing to maternal Se deficiency was observed in the liver of female offspring during weaning according to the factorial analysis (*p* < 0.001). Meanwhile, GDM induced an increase in GSH-Px activity in the liver of male offspring during weaning (*p* = 0.004); however, Se deficiency in maternal mice decreased GSH-Px (*p* < 0.001) and SOD activity (*p* = 0.001).

For the liver of adult female offspring, GSH-Px activity in the LSe group was significantly lower than that in the CON and GDM groups; GSH-Px activity in the LSe-GDM group was significantly lower than that in the GDM group, and that in the LSe group was significantly lower than the LSe-GDM group. SOD activity in the LSe and LSe-GDM groups was significantly lower than that in the CON and GDM groups (Figure 2E,F). GSH-Px activity in the LSe group was significantly lower than that in the CON, GDM, and LSe-GDM groups in the liver of adult male offspring (Figure 2G).

The GDM resulted in increased liver GSH-Px activity for adult female offspring based on factorial analysis (*p* < 0.001); Se deficiency in maternal mice led to a decrease in GSH-Px (*p* < 0.001) and SOD activities (*p* = 0.005); the interaction between GDM and Se deficiency impacted GSH-Px activity (*p* < 0.001) (Figure 2E). Meanwhile, GDM resulted in increased GSH-Px activity in the liver of adult male offspring (*p* < 0.001); Se deficiency in maternal mice decreased GSH-Px activity (*p* < 0.001); and the interaction between GDM and Se deficiency had an impact on GSH-Px activity (*p* < 0.001) (Figure 2G). There was no difference in MDA levels in any of the mice, including maternal and offspring mice (Supplementary Figure S3). The GSH-Px and SOD of maternal mice are shown in Supplementary Figure S4.

### 3.4. Effects of GDM and Se Deficiency on the PI3K/Akt Signaling Pathway-Related Proteins in Offspring

**Figure 3:**
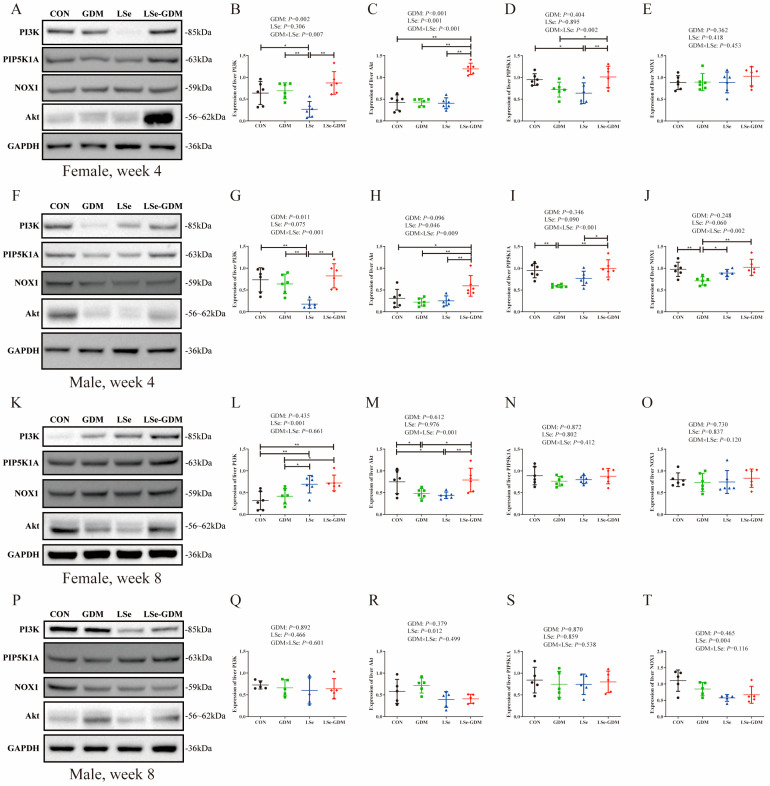
The effects of GDM and Se deficiency on PI3K/Akt signaling pathway-related proteins in the liver of offspring. (**A**) The protein expression levels of the PI3K/Akt signaling pathway in the liver of weaning female offspring; (**B**) The protein expression levels of PI3K in the liver of weaning female offspring; (**C**) The protein expression levels of Akt in the liver of weaning female offspring; (**D**) The protein expression levels of PIP5K1A in the liver of weaning female offspring; (**E**) The protein expression levels of NOX1 in the liver of weaning female offspring; (**F**) The protein expression levels of the PI3K/Akt signaling pathway in the liver of weaning male offspring; (**G**) The protein expression levels of PI3K in the liver of weaning male offspring; (**H**) The protein expression levels of Akt in the liver of weaning male offspring; (**I**) The protein expression levels of PIP5K1A in the liver of weaning male offspring; (**J**) The protein expression levels of NOX1 in the liver of weaning male offspring; (**K**) The protein expression levels of the PI3K/Akt signaling pathway in the liver of adult female offspring; (**L**) The protein expression levels of PI3K in the liver of adult female offspring; (**M**) The protein expression levels of Akt in the liver of adult female offspring; (**N**) The protein expression levels of PIP5K1A in the liver of adult female offspring; (**O**) The protein expression levels of NOX1 in the liver of adult female offspring; (**P**) The protein expression levels of the PI3K/Akt signaling pathway in the liver of adult male offspring; (**Q**) The protein expression levels of PI3K in the liver of adult male offspring; (**R**) The protein expression levels of Akt in the liver of adult male offspring; (**S**) The protein expression levels of PIP5K1A in the liver of adult male offspring; (**T**) The protein expression levels of NOX1 in the liver of adult male offspring; * indicate *p* < 0.05, ** indicate *p* < 0.01.

The effects of GDM and Se deficiency on the expression level of PI3K/Akt pathway proteins in the liver of weaning female offspring are shown in Figure 3A. The expression level of the PI3K protein in the LSe group was significantly lower than that in the CON, GDM and LSe-GDM groups (Figure 3B); the expression level of the Akt protein in the LSe-GDM group was significantly higher than that in the CON, GDM and LSe groups (Figure 3C); the expression level of the PIP5K1A protein in the LSe group was significantly lower than that in the CON and LSe-GDM groups, and that in the GDM group was significantly lower than the LSe-GDM group (Figure 3D). No differences were observed in the protein expression levels of NOX1 among the four groups (Figure 3E). The impacts of GDM and Se deficiency on the expression level of the PI3K/Akt pathway protein in the liver of weaning male offspring are shown in Figure 3F. the expression level of the PI3K protein in the LSe group was significantly lower than that in the CON, GDM and LSe-GDM groups (Figure 3G); the expression level of the Akt protein in the LSe-GDM group was significantly higher than that in the CON, GDM and LSe groups (Figure 3H); the expression level of the PIP5K1A protein in the GDM group was significantly lower than that in the CON and LSe-GDM groups, and that in the LSe group was significantly lower than the LSe-GDM group (Figure 3I). The expression level of the NOX1 protein in the GDM group was significantly lower than that in the CON, LSe, and LSe-GDM groups (Figure 3J).

Factorial analysis showed that GDM increased the protein expression levels of PI3K (*p* = 0.002) and Akt (*p* < 0.001) but had no effect on the protein expression levels of PIP5K1A and NOX1 in the livers of weaning female offspring; Se deficiency in maternal mice diminished the Akt protein expression level (*p* < 0.001) but failed to exert any effect on the protein expression levels of PI3K, PIP5K1A, and NOX1. Furthermore, the interaction between GDM and Se deficiency affected the protein expression levels of PI3K (*p* = 0.007), Akt (*p* < 0.001) and PIP5K1A (*p* = 0.002) but had no effect on the protein expression level of NOX1 (Figure 3B–E). In the livers of male offspring during weaning, GDM increased the protein expression level of PI3K (*p* = 0.001) but had no impact on the protein expression levels of Akt, PIP5K1A, and NOX1. Se deficiency in maternal mice influenced the protein expression level of Akt (*p* = 0.046) but had no effect on the protein expression levels of PI3K, PIP5K1A, and NOX1. The interaction between GDM and Se deficiency affected the protein expression levels of PI3K (*p* = 0.001), Akt (*p* = 0.009), PIP5K1A (*p* < 0.001), and NOX1 (*p* = 0.003) (Figure 3G–J).

The effect of GDM and Se deficiency on the expression level of PI3K/Akt pathway proteins in the liver of adult female offspring is shown in Figure 3K. The expression level of the PI3K protein in the LSe and LSe-GDM groups was significantly higher than that in the CON and GDM groups (Figure 3L); the expression level of the Akt protein in the CON group was significantly higher than that in the GDM and LSe groups, and that in the LSe-GDM group was significantly higher than the GDM and LSe groups (Figure 3M). No differences were observed in the expression levels of PIP5K1A or NOX1 among the four groups (Figure 3N,O). There was no difference in the protein expression levels of PI3K, Akt, PIP5K1A, and NOX1 among the four groups in the liver of adult male offspring (Figure 3P–T).

Factorial analysis revealed that GDM had no effect on the protein expression levels of PI3K, Akt, PIP5K1A, and NOX1 in the liver of adult female offspring; Se deficiency in maternal mice increased the protein expression level of PI3K (*p* < 0.001) but had no effect on the protein expression levels of Akt, PIP5K1A, and NOX1. Furthermore, the interaction between GDM and Se deficiency affected the protein expression level of Akt (*p* = 0.001) but failed to exert any influence on the protein expression levels of PI3K, PIP5K1A, and NOX1 (Figure 3L–O). A deficiency in GDM and Se had no effect on the protein expression levels of the PI3K/Akt pathway in the liver of adult male offspring. The effects of GDM and Se deficiency on PI3K/Akt signaling pathway-related proteins in mothers are shown in Supplementary Figure S5.

## 4. Discussion

S961 is a short-acting insulin receptor antagonist. After the cessation of drug administration, blood glucose levels returned to normal after a period of time; this response is more similar to human GDM and is a more reasonable approach than a streptozotocin injection. Some studies have shown that the order of body Se levels in mouse tissues from highest to lowest is kidney > heart > liver [23]. Thus, the Se content in the body is represented by the Se content in the kidneys. In this study, the Se content in the kidneys of pregnant mice for the two groups with Se deficiency was significantly lower than that in the two groups fed a basic diet.

The differences in the birth weight of the offspring could indicate that maternal Se deficiency and GDM affects the nutrient intake of the fetus in the uterus, limiting its growth and development. After birth, the offspring were fed by the mother until weaning, and the weight of the offspring in the GDM group was significantly higher than that in the non-GDM group. This finding is consistent with the conclusion that intrauterine exposure to GDM is an important risk factor for childhood obesity [24]. The body weights of female offspring in the LSe group after weaning until adulthood were significantly lower than those in the other three groups. However, the impact of maternal GDM and Se deficiency on female offspring weakened, and female offspring in the CON group had the highest body weight as the offspring independently consumed a basic diet. The body weight of male offspring in the GDM and LSe-GDM groups was significantly higher than that in the LSe group, and it remained the highest in the GDM group; the body weight of offspring of the LSe group was consistently the lowest (regardless of sex). This indicates that Se deficiency in maternal mice affect the growth and development of offspring. This finding is consistent with those of previous results [25]. Early nutritional deficiencies were found to still exist in adulthood even if the offspring left the Se-deficient environment in later stages. This study found sex differences in the impact of maternal GDM on the adult body weight of offspring. Maternal GDM can significantly increase the weight of adult male offspring; however, its effect on adult female offspring is insignificant. This suggests that maternal GDM can increase the susceptibility of male offspring to obesity in adulthood. Many previous investigations have shown that male offspring exposed to GDM have more fat during school age and a higher BMI and obesity risk during childhood, adolescence, and early adulthood compared to those in non-GDM groups. This is consistent with the results of this study [26,27].

Visceral organs are important components of the body that continuously develop and mature as animals grow. Changes in their mass, volume, and structure reflect their growth, development, and health status [28,29]. The organ coefficient is the ratio of the weight of a certain organ in an experimental animal compared to its body weight. Normally, the organ-to-body weight ratio remains relatively constant. However, changes in weight can damage organs; therefore, the organ coefficient also changes accordingly. This study found that maternal GDM affected the development of various organs in female offspring during weaning. Both maternal GDM and Se deficiency had an impact on organ development in male offspring during weaning, and their effects on different organs varied. After weaning, all offspring were fed a basic feed, and there were no differences in organ coefficients between the four groups of adult female and male offspring. This emphasizes that female and male offspring are free from Se deficiency and GDM during their growth period and that postnatal nutritional supplementation can help them catch up with growth and improve developmental deficiencies in early childhood.

This study found that Se deficiency in maternal mice affected the glucose metabolism of female offspring during their growth period; however, maternal GDM affected glucose metabolism in adult female offspring. The effect of maternal GDM and Se deficiency on male offspring decreased with environmental changes until they disappeared in adulthood. There were differences regarding the effects of maternal GDM and Se deficiency on glucose metabolism in offspring based on their sex. Maternal GDM or Se deficiency increased the risk of abnormal glucose metabolism in female offspring during childhood and adulthood but had a relatively small impact on glucose metabolism in male offspring. Previous studies have found that the thickness of skin folds, blood sugar, and insulin resistance in the daughters of mothers with diabetes in childhood are significantly higher than those of sons, which is consistent with the results of this study [30]. Meanwhile, the outcomes of the present study are consistent with those of Pierre Hofstee during weaning [31].

The results of oxidative stress in the maternal liver and placenta in this study are included in the Supplementary Materials. GSH-Px activity in the placenta was similar to that in the liver; however, SOD activity in the placenta was different from that in the liver. This indicates that the placenta has a barrier against oxidative damage, thereby reducing and protecting against the damage caused by maternal GDM in fetal mice [32,33]. During the weaning period, the liver GSH-Px activity of female offspring from the two groups of Se-deficient maternal mice was significantly lower than that in the two groups of normal Se maternal mice. After weaning, the environment of GDM and Se deficiency improved; however, GSH-Px activity in the livers of the LSe group remained low. This indicates that the effect of Se deficiency on female offspring persists until adulthood and is not easily replenished on the next day, which is consistent with previous results [34,35,36]. The effect of maternal Se deficiency on SOD activity in female offspring was insignificant in the early stages; however, it exhibited long-term effects as the offspring grew and developed. The effects of maternal GDM and Se deficiency on GSH-Px activity in the livers of male offspring were consistent with those of female offspring; however, the impact of SOD activity in males was opposite to that in females. The effect of maternal Se deficiency on SOD activity in male offspring was short-term.

There were differences in the expression of PI3K/Akt signaling pathway-related proteins between female offspring during weaning and adulthood. The trends in PI3K and Akt protein levels were consistent with the blood glucose performance of adult females. Studies on Akt-knockout animals have found that the knockout group shows symptoms such as insulin resistance, hyperglycemia, and hyperinsulinemia [37]. The results of this study are similar to those of the elimination group. The blood sugar levels of offspring with reduced Akt expression were higher than those in other populations. The expression of PI3K/Akt signaling pathway-related proteins in the liver of male offspring during weaning was influenced by the interaction between maternal GDM and Se deficiency. After weaning, the effects of maternal GDM and Se deficiency on male offspring decreased with environmental changes until they disappeared in adulthood. This is consistent with the results of adult male blood glucose. The protein expression results of adult female and male offspring in this study were consistent with the blood glucose levels. This indicates that the mechanism through which GDM and Se deficiency affect glucose metabolism in female offspring is related to the PI3K/Akt signaling pathway. In addition, we performed pathological HE staining on the liver and pancreas tissues of pregnant mice and their offspring, the specific results of which are shown in Supplementary Figures S6–S8. 

This study suggests that maternal GDM and Se deficiency in early life can reduce the activities of GSH-Px and SOD in the liver and placenta of pregnant mice, enhance oxidative stress, and inhibit the expression of PI3K/Akt signaling pathway-related proteins in the liver and placenta, thereby worsening glucose metabolism in pregnant mice. The liver is the main organ for glucose metabolism, and the placenta is the bridge between mother and fetus. When oxidative stress and metabolic abnormalities occur in the liver and placenta, fetal nutrient intake in utero is affected, and their intrauterine growth and development are restricted, resulting in a low birth weight.

Previous studies have reported slight sex-related differences in the progression of metabolic diseases in Se-deficient offspring [38]. These studies focused on the effect of Se deficiency during pregnancy and on the movement of offspring, which differed from the measurement indicators used in this study. Nevertheless, it provides insights into a possible mechanism for future research on the effects of Se deficiency on the offspring of different sexes. In humans, fluctuations in selenoprotein expression and activity are typically not as significant as those observed in animal studies [39]. This study did not include population studies, and collecting blood and placental samples from the population in the future could help to propose scientific hypotheses. This study further highlights the importance of obtaining sufficient micronutrients during pregnancy and the early postpartum period, adding to the growing body of evidence in this field. Cellular studies show that micronutrient supplementation can increase micronutrient content in the placenta [40]. Optimal maternal micronutrient levels are crucial for placental health and provide novel ideas for promoting the healthy growth of offspring in GDM and Se-deficient mothers.

## 5. Conclusions

GDM and maternal Se deficiency decreased fetal weight by exposing fetuses to hyperglycemia or oxidative stress in the early stages of life. GDM increased susceptibility to obesity in lactating offspring, and there was a sex difference. There was also a sex difference in the effects of GDM and Se deficiency on glucose metabolism and the expression of PI3K/Akt signaling pathway-related proteins in offspring. After weaning, the impact on females continued until adulthood, but for males, these disappeared during adulthood. This study provides evidence that emphasizes the need to obtain sufficient micronutrients during pregnancy and early postpartum while also providing new ideas for clinical treatment or the prevention of GDM and Se deficiency.

## Figures and Tables

**Table 1:** The fasting blood glucose of offspring (mmol/L) (Mean ± SD).

Week		CON	GDM	LSe	LSe-GDM	*p*
	Female					
4		3.40 ± 1.04 ^ac^	5.33 ± 1.33	3.27 ± 0.53 ^bd^	5.58 ± 1.31	**0.001**
5		4.25 ± 1.05	5.05 ± 0.65	5.58 ± 0.76	4.75 ± 0.97	0.092
6		4.50 ± 0.77	5.32 ± 0.62	5.33 ± 1.26	4.65 ± 1.01	0.307
7		4.53 ± 0.70 ^e^	5.33 ± 1.30	6.28 ± 0.47	5.30 ± 0.72	**0.018**
8		3.93 ± 0.40 ^a^	5.43 ± 1.22	4.46 ± 0.95	4.77 ± 0.69	**0.049**
	Male					
4		5.87 ± 0.88	6.07 ± 1.62	3.25 ± 1.10 ^be^	4.68 ± 1.51	**0.005**
5		5.48 ± 0.79	5.43 ± 1.43	4.92 ± 1.02	5.37 ± 0.58	0.754
6		5.65 ± 0.80	5.80 ± 0.83	4.93 ± 1.51	5.35 ± 0.72	0.482
7		5.68 ± 0.90	6.15 ± 1.26	5.25 ± 1.09	6.23 ± 0.25	0.281
8		5.67 ± 0.74	6.07 ± 1.30	5.24 ± 1.22	5.38 ± 0.67	0.543

CON, basic diet + PBS injection; GDM, basic diet + S961 injection; LSe, low Se-diet + PBS injection; LSe-GDM, low-Se diet + S961 injection; ^a, b, c, d, e^ indicates *p* < 0.05 ^a^ GDM vs. CON, ^b^ GDM vs. LSe, ^c^ LSe-GDM vs. CON, ^d^ LSe-GDM vs. LSe, ^e^ LSe vs. CON; N_CON_ = 6, N_GDM_ = 6, N_LSe_ = 6, N_LSe-GDM_ = 6. Bold indicates *p* < 0.05 among the four groups.

## Data Availability

Our data are confidential for some projects, and we will consider making it public when all our projects are complete.

## Supplementary Materials

The following supporting information can be downloaded at: https://www.mdpi.com/article/10.3390/nu15214519/s1, Table S1: Composition of the basic diet; Table S2: Preparation of Se standard curve; Table S3: The antibodies of Western blot; Figure S1: The baseline and postpartum blood glucose levels in maternal mice; Figure S2: The establishment of GDM and Se deficiency animal models; Figure S3: The MDA of maternal and offspring; Figure S4: The GSH-Px and SOD of maternal; Figure S5: The effects of GDM and Se deficiency on the PI3K/Akt signaling pathway-related proteins in maternal liver and placenta. Figure S6: The HE section of maternal mice tissue. Figure S7: The HE section of offspring mice tissue in the 4th week. Figure S8: The HE section of offspring mice tissue in the 8th week.
