# Supplementary material for: Effects of Gestational Diabetes Mellitus and Selenium Deficiency on the Offspring Growth and Blood Glucose Mechanisms of C57BL/6J Mice
Source: Nutrients. 2023 Oct 25;15(21):4519. doi: 10.3390/nu15214519 (PMC10647445; doi:10.3390/nu15214519)
Supplement: Supplementary file 1 [file nutrients-15-04519-s001.zip › nutrients-2620349-supplementary.pdf]

Supplementary Table S1 Composition of the basic diet

Ingredients	Basic diet	
	Weight (g)	Energy (kcal)
Casein	189.58	758.32
Cystine	2.84	11.36
Corn starch	298.59	1194.36
Maltodextrin	33.18	132.72
Sucrose	331.77	1327.08
Cellulose	47.40	0
Soybean oil	23.70	213.30
Lard oil	18.96	170.64
Mineral mix	9.48	0
Calcium bicarbonate	12.32	0
Calcium carbonate	5.21	0
Calcium citrate	15.64	0
Vitamin mix	9.48	37.92
Choline bitartrate	1.90	0
Total	1000	3845.70

Supplementary Table S2 Preparation of Se standard curve

	Standard Application Solution (mL)	HCL (mL)	Concentration (μg/L)	To 10 (mL)
1	0.00	0.5	0	10
2	0.05	0.5	5	10
3	0.10	0.5	10	10
4	0.20	0.5	20	10
5	0.40	0.5	40	10
6	0.80	0.5	80	10

Supplementary Table S3 The antibodies of Western blot

Antibodies	Dilutability
GAPDH	1:10000
B2M	1:10000
PI3K	1:10000
Akt	1:5000
NOX1	1:5000
PIP5K1A	1:10000
Goat anti-Mouse IgG (H&L)-HRP	1:5000
Goat anti-Rabbit IgG (H&L)-HRP	1:5000

Establishment of GDM and Se Deficiency Animal Models

No difference was observed in the FBG levels of baseline and GD 7 among the four groups of maternal mice (Supplementary Figure S1A-B). On GD 14, the OGTT results suggested that the blood glucose levels in the GDM and LSe-GDM groups were significantly higher than those in the CON and LSe groups at 30, 60, 120, and 180 min; and at 180 min, the blood glucose level in the LSe-GDM group was higher than that in the GDM group (Supplementary Figure S2A). The area under curve (AUC) of blood glucose in the GDM and LSe-GDM groups was significantly higher than that in the CON and LSe groups (Supplementary Figure S2B). On GD 18, the fasting insulin levels in the GDM and LSe-GDM groups were significantly higher than that in the CON group, and that in the GDM group was significantly higher than that in the LSe group (Supplementary Figure S2C). Subcutaneous injection of S961 alone or in combination

with low Se diet did not lead to death. According to the OGTT and AUC of blood glucose, it has been proven that all pregnant mice injected with S961 developed insulin resistance, and a GDM animal model was successfully established. The OGTT after 4 weeks postpartum showed no difference in blood glucose levels among the four groups (Supplementary Figure S1C), which indicated that the maternal blood glucose returned to normal after a period of postpartum, which was in line with the characteristics of GDM.

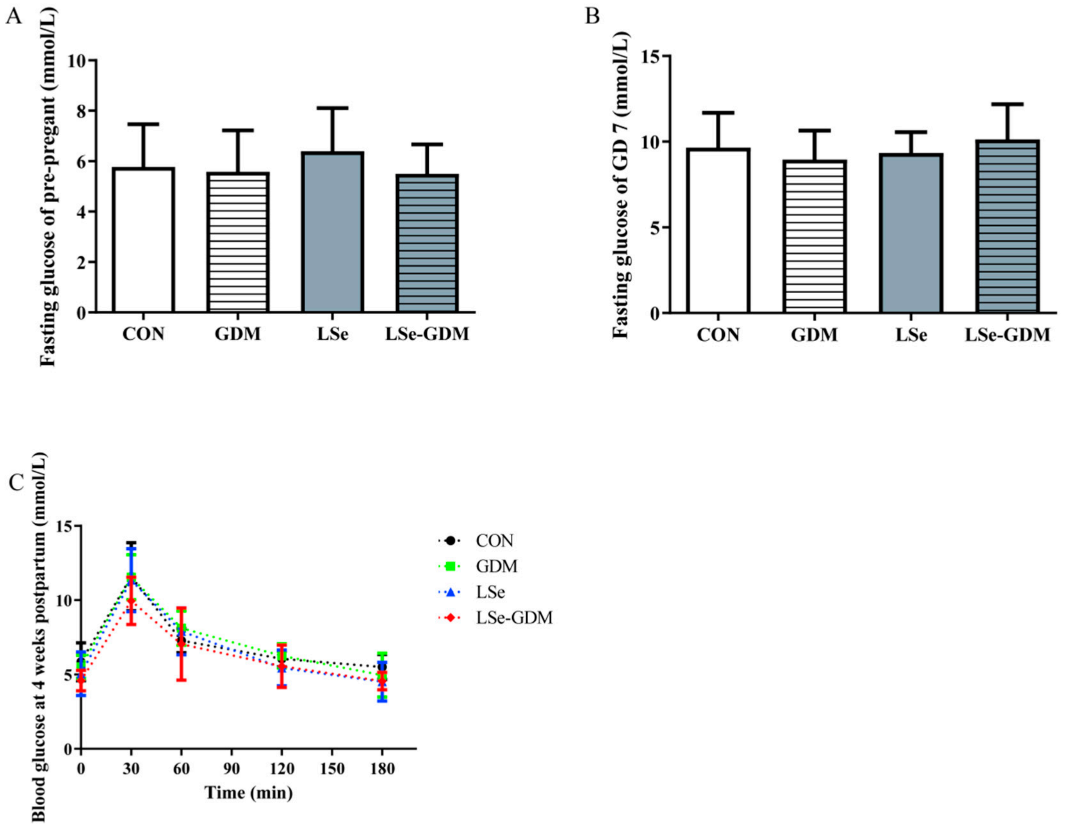

Supplementary Figure S1. The baseline and postpartum blood glucose levels in maternal mice

A. The fasting glucose of pre-pregnant; B. The fasting glucose of GD 7; C. The OGTT of 4 weeks postpartum; CON, basic diet + PBS injection; GDM, basic diet + S961 injection; LSe, low Se diet + PBS injection; LSe-GDM, low Se diet + S961 injection.

The Se content in the kidneys of pregnant mice in the low Se diet group was significantly lower than in the basic diet group. Factorial analysis suggested that feeding low Se diet decreased Se content (P<0.001), and a Se deficiency model was successfully established (Supplementary Figure S2D).

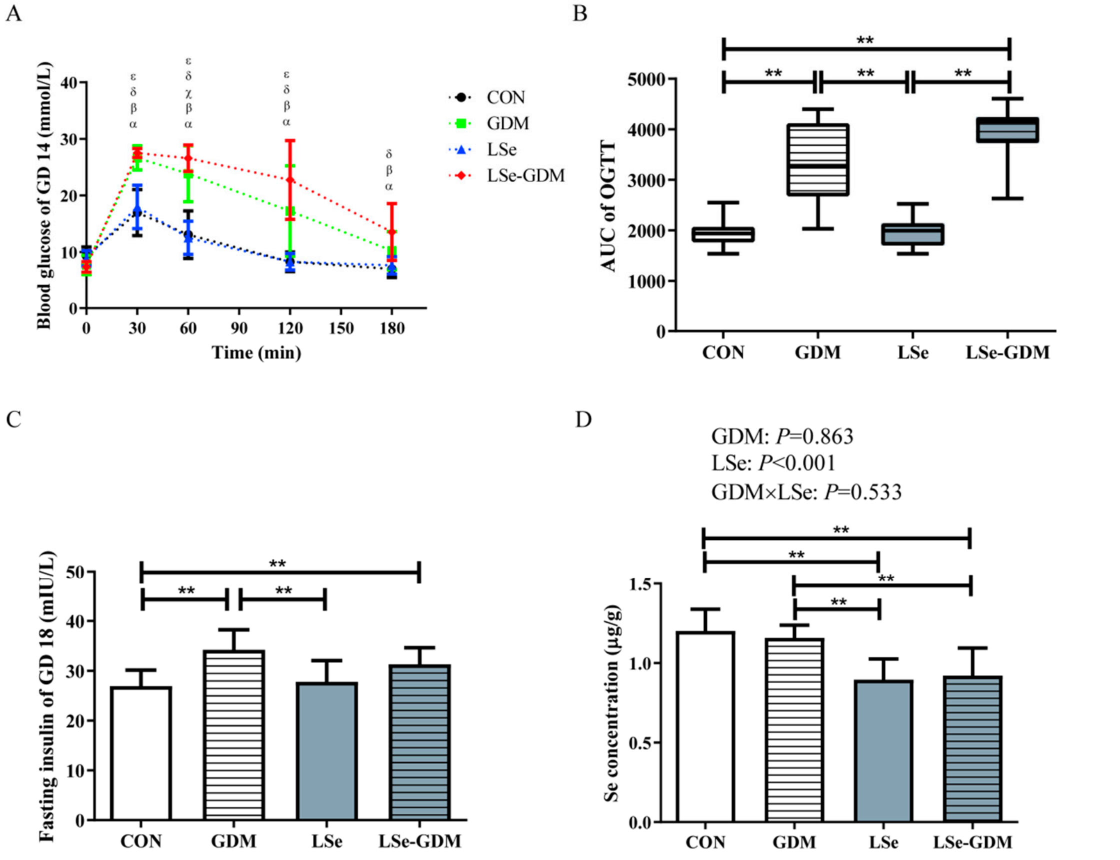

Supplementary Figure S2. The establishment of GDM and Se deficiency animal models

A. The OGTT of GD 14 (N_{CON}=18, N_{GDM}=16, N_{LSe}=18, $N_{LSe\text{-}GDM}$=18); B. AUC of OGTT on GD 14 (N_{CON}=18, N_{GDM}=16, N_{LSe}=18, $N_{LSe\text{-}GDM}$=18); C. Fasting insulin of GD 18 (N_{CON} = 12, N_{GDM} = 8, N_{LSe} = 11, $N_{LSe\text{-}GDM}$ = 12); D. The Se concentration of pregnant mice (N_{CON} = 12, N_{GDM} = 8, N_{LSe} = 11, $N_{LSe\text{-}GDM}$ = 12); $^{\alpha,\ \beta,\ \chi,\ \delta,\ \varepsilon}$ indicate $P < 0.05$ $^{\alpha}$ GDM vs. CON, $^{\beta}$ GDM vs. LSe, $^{\chi}$ LSe-GDM vs. CON, $^{\delta}$ LSe-GDM vs. LSe, $^{\varepsilon}$ LSe-GDM vs. GDM; ** indicate $P < 0.01$; CON, basic diet + PBS injection; GDM, basic diet + S961 injection; LSe, low Se diet + PBS injection; LSe-GDM, low Se diet + S961 injection.

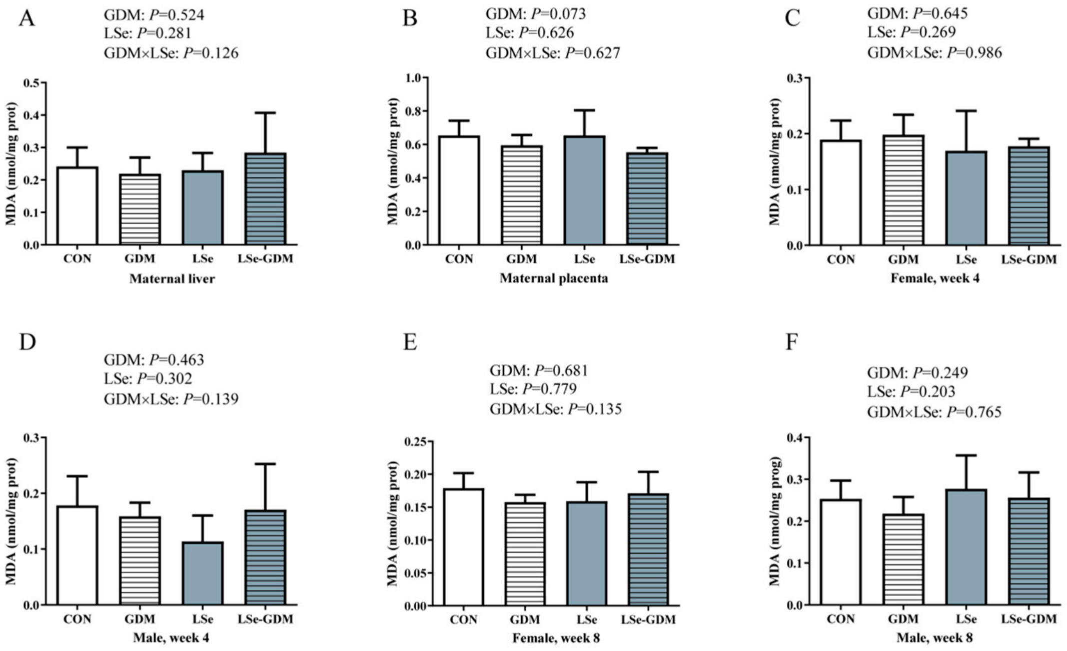

Supplementary Figure S3. The MDA of maternal mice and offspring

A. The MDA of maternal liver; B. The MDA of maternal placenta; C. The MDA of female offspring in the 4th week; D. The MDA of male offspring in the 4th week; E. The MDA of female offspring in the 8th week; F. The MDA of male offspring in the 8th week; CON, basic diet + PBS injection; GDM, basic diet + S961 injection; LSe, low Se diet + PBS injection; LSe-GDM, low Se diet + S961 injection.

The Effects of GDM and Se Deficiency on Oxidative Stress in Maternal Mice

For pregnant mice, the liver GSH-Px activities in the LSe and LSe-GDM groups were significantly lower than those in the CON and GDM groups. The liver SOD activities in the CON and LSe groups were significantly higher than that in the GDM group, but the LSe-GDM group was significantly higher than that in the GDM group (Supplementary Figure S4A-B). The placenta GSH-Px activity in the LSe group was significantly lower than those in the CON and GDM groups, and the LSe-GDM group was significantly lower than that in the CON group. In addition, no difference was

observed in placenta SOD activity among the four groups (Supplementary Figure S4C-D).

For pregnant mice, factorial analysis suggested that Se deficiency decreased the liver GSH-Px (*P*<0.001) and increased the liver SOD activity (*P*=0.009). GDM decreased liver SOD activity (*P*=0.017), however, the interaction between GDM and Se deficiency had no effect on the GSH-Px and SOD activity in the liver (Supplementary Figure S4A-B). Se deficiency decreased the placenta GSH-Px activity (*P*=0.001), however, the interaction between GDM alone and low Se fed had no effect on the GSH-Px and SOD activity in the placenta (Supplementary Figure S4C-D).

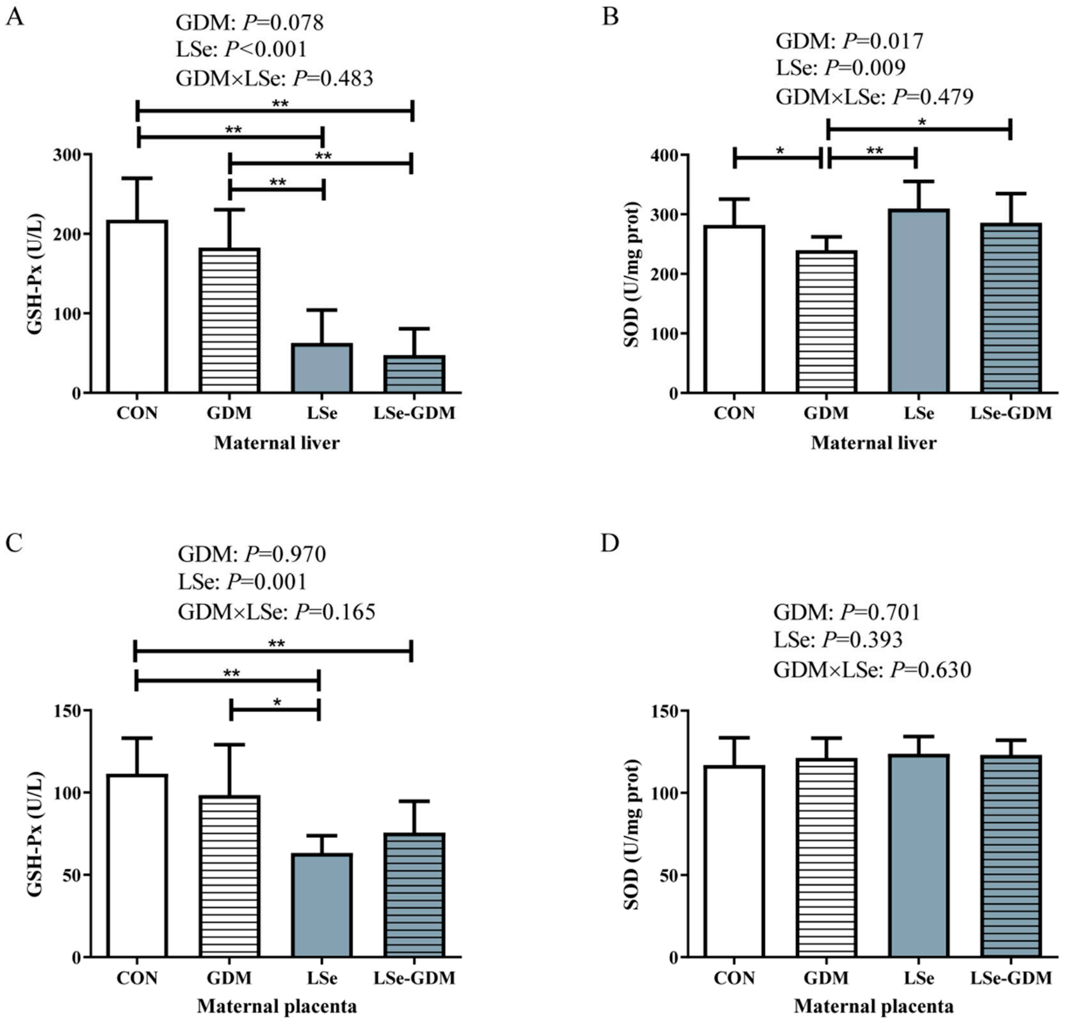

Supplementary Figure S4. The GSH-Px and SOD of maternal mice

A. The GSH-Px of maternal liver; B. The SOD of maternal liver; C. The GSH-Px of maternal placenta; D. The SOD of maternal placenta.

The Effects of GDM and Se deficiency on the PI3K/Akt signaling pathway-related proteins in maternal mice

GDM and Se deficiency affected the expression levels of the PI3K/Akt signaling pathway-related proteins in the liver of pregnant mice (Supplementary Figure S5A). In the detail, the PI3K protein expression level in the GDM group was significantly lower than those in the CON and LSe-GDM groups, and that in the LSe group was significantly lower than that in the LSe-GDM group (Supplementary Figure S5B); the expression level of Akt protein in the GDM group was significantly lower than those in the CON and LSe-GDM groups, and that in the LSe group was significantly lower than that in the LSe-GDM group (Supplementary Figure S5C). No difference was observed in the expression levels of PIP5K1A and NOX1 proteins among the four groups (Supplementary Figure S5D-E). GDM and Se deficiency impacted the expression level of the PI3K/Akt signaling pathway proteins in the placenta of pregnant mice (Supplementary Figure S5F), the expression level of PI3K protein in the GDM group was significantly lower than those in the CON, LSe, and LSe-GDM groups (Supplementary Figure S5G); the expression level of Akt protein in the LSe group was significantly higher than those in the CON, GDM, and LSe-GDM groups (Supplementary Figure S5H); the expression level of PIP5K1A protein in the GDM group was significantly lower than those in the CON and LSe groups (Supplementary

Figure S5I). No difference was observed in the expression level of NOX1 protein among the four groups (Supplementary Figure S5J).

For the liver of pregnant mice, factorial analysis indicated that the interaction between GDM and Se deficiency affected the protein expression levels of PI3K (*P*=0.001) and Akt (*P*=0.001) but had no effect on the protein expression levels of PIP5K1A and NOX1. GDM or Se deficiency alone did not impact the protein expression levels of PI3K, Akt, PIP5K1A, and NOX1 (Supplementary Figure S5B-E). For the placenta of pregnant mice, GDM reduced the protein expression levels of PI3K (*P*=0.010), Akt (*P*=0.015), and PIP5K1A (*P*=0.009) but had no effect on the protein expression level of NOX1. Se deficiency increased the protein expression level of Akt (*P*=0.014), but had no difference on the protein expression levels of PI3K, Akt, and NOX1. The interaction between GDM and Se deficiency did not affect the protein expression levels of PI3K, Akt, PIP5K1A, and NOX1 (Supplementary Figure S5G-J).

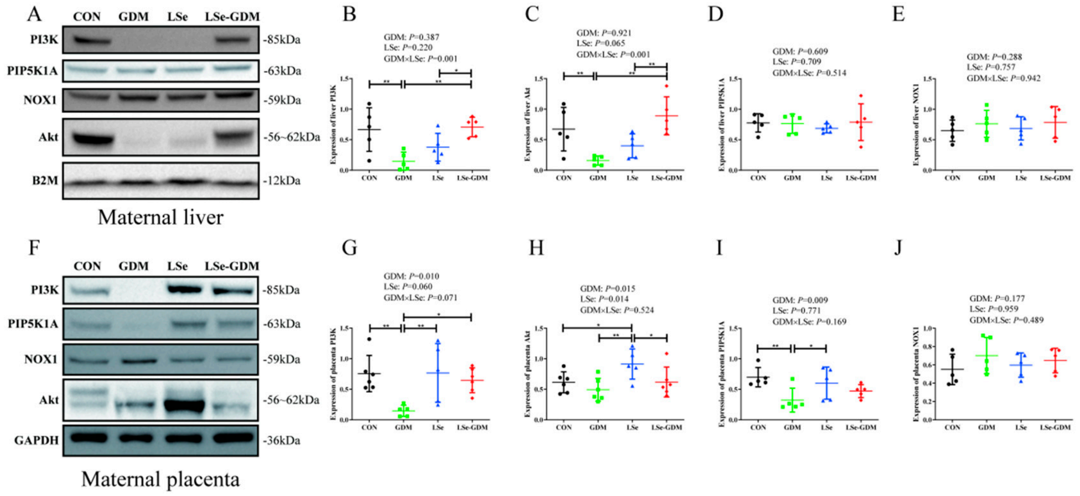

Supplementary Figure S5. The effects of GDM and Se deficiency on the PI3K/Akt signaling pathway-related proteins in maternal liver and placenta

A. The protein expression levels of the PI3K/Akt signaling pathway in maternal liver; B. The protein expression levels of PI3K in maternal liver; C. The protein expression levels of Akt in maternal liver; D. The protein expression levels of PIP5K1A in maternal liver; E. The protein expression levels of NOX1 in maternal liver; F. The protein expression levels of the PI3K/Akt signaling pathway in maternal placenta. G. The protein expression levels of PI3K in maternal placenta; H. The protein expression levels of Akt in maternal placenta; I. The protein expression levels of PIP5K1A in maternal placenta; J. The protein expression levels of NOX1 in maternal placenta.

The HE section tissue of maternal mice and offspring

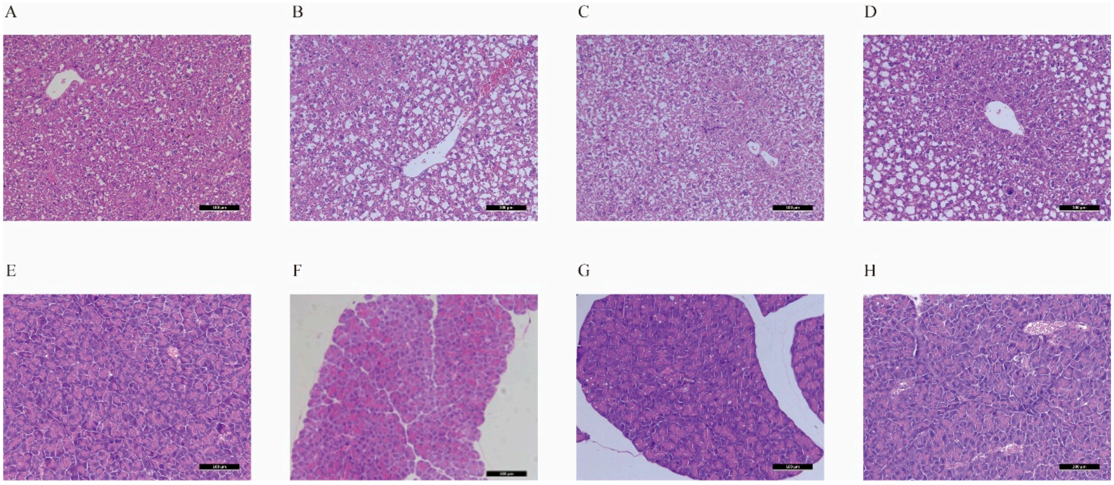

Supplementary Figure S6. The HE section of maternal mice tissue

A. The liver of CON; B. The liver of GDM; C. The liver of LSe; D. The liver of LSe-GDM; E. The pancreas of CON; F. The pancreas of GDM; G. The pancreas of LSe; H. The pancreas of LSe-GDM.

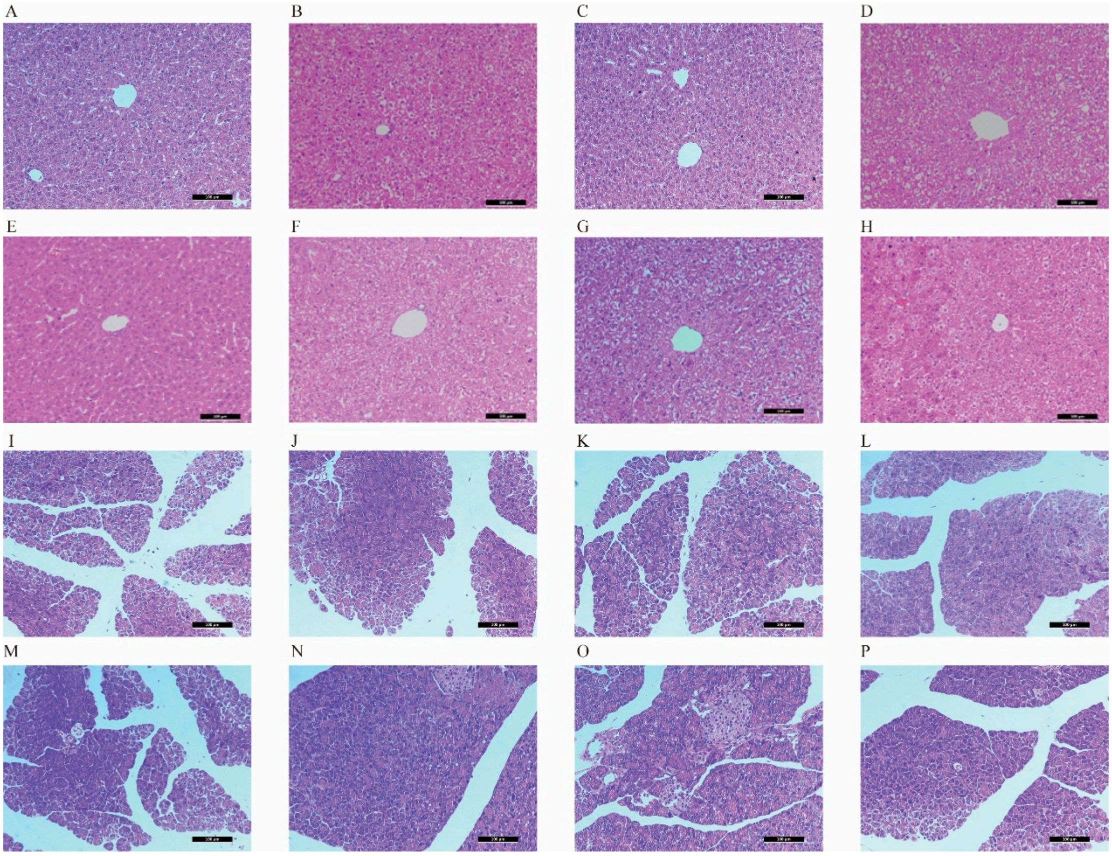

Supplementary Figure S7 The HE section of offspring mice tissue at the 4th week

A. The female liver of CON; B. The female liver of GDM; C. The female liver of LSe; D. The female liver of LSe-GDM; E. The male liver of CON; F. The male liver of GDM; G. The male liver of LSe; H. The male liver of LSe-GDM; I. The female pancreas of CON; J. The female pancreas of GDM; K. The female pancreas of LSe; L. The female pancreas of LSe-GDM; M. The male pancreas of CON; N. The male pancreas of GDM; O. The male pancreas of LSe; P. The male pancreas of LSe-GDM.

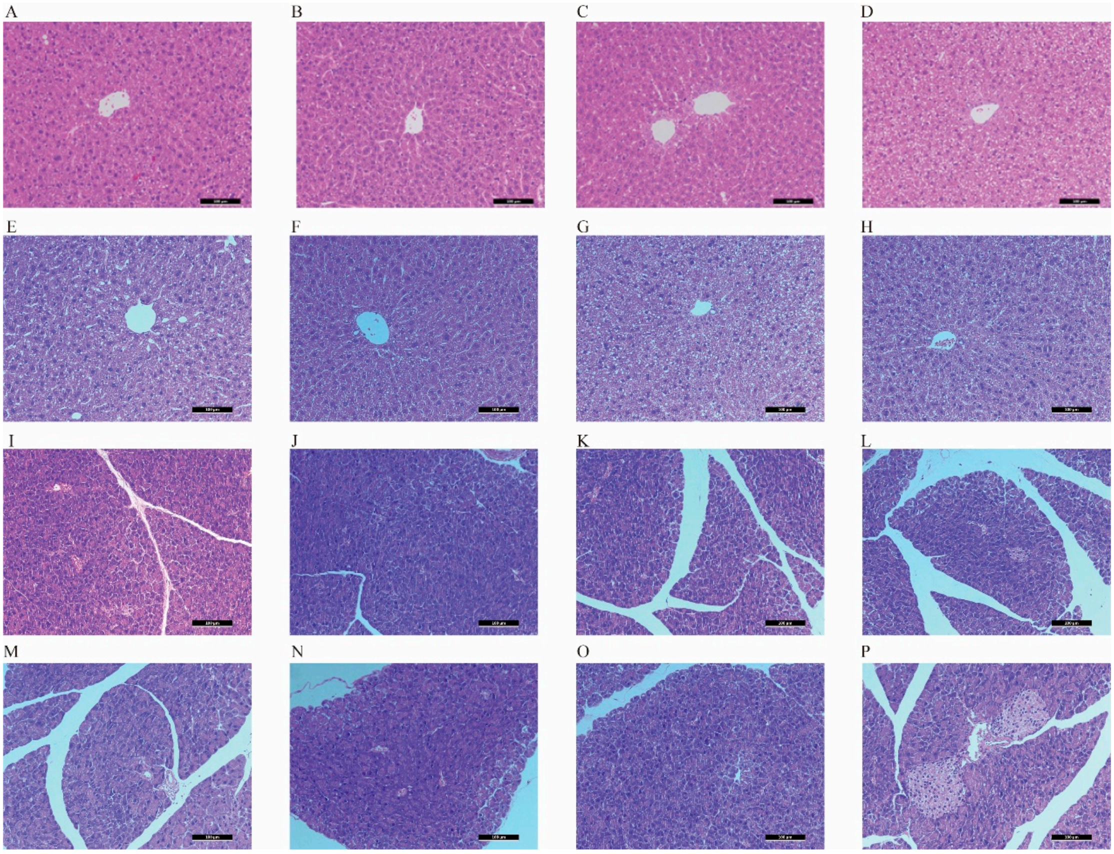

Supplementary Figure S8 The HE section of offspring mice tissue at the 8th week

A. The female liver of CON; B. The female liver of GDM; C. The female liver of LSe; D. The female liver of LSe-GDM; E. The male liver of CON; F. The male liver of GDM; G. The male liver of LSe; H. The male liver of LSe-GDM; I. The female pancreas of CON; J. The female pancreas of GDM; K. The female pancreas of LSe; L. The female pancreas of LSe-GDM; M. The male pancreas of CON; N. The male pancreas of GDM; O. The male pancreas of LSe; P. The male pancreas of LSe-GDM.

The experimental instruments and materials

Experimental instruments and materials	Source
C57Bl/6J	Skbex Biotechnology, Henan, China
basic diet (H10010)	Huafukang Bioscience Co., Ltd, Beijing, China
low Se diet (Special customization)	Huafukang Bioscience Co., Ltd, Beijing, China
S961	Jiepeptide Biotechnology Co., Ltd., Nanjing, China
GSH-Px test kit	Nanjing Jiancheng Bioengineering Institute, Nanjing, China
SOD test kit	Nanjing Jiancheng Bioengineering Institute, Nanjing, China
MDA test kit	Nanjing Jiancheng Bioengineering Institute, Nanjing, China
BCA test kit	Beyotime Biotechnology, China
FuturePAGE^{TM}	Nanjing ACE Biotechnology Co., Ltd, Nanjing, China
PVDF membranes	Merck Millipore Ltd, Ireland
Wet transfer unit	Bio-Rad Laboratories, Inc., Hercules, CA
Enhanced chemiluminescence substrate reactions	Meilun Biotechnology Co., Ltd, Dalian, China
PI3K antibody	60225-1-lg, Proteintech Group
Akt antibody	60203-2-lg, Proteintech Group
NOX1 antibody	17772-1-AP, Proteintech Group
PIP5K1A antibody	15713-1-AP, Proteintech Group
